# Co-occurrence of carbapenemase encoding genes in *Acinetobacter baumannii*, a dream or reality?

**DOI:** 10.1186/s12866-018-1252-2

**Published:** 2018-09-05

**Authors:** Linda Hadjadj, Sofiane Bakour, Jean-Marc Rolain

**Affiliations:** Aix Marseille Univ, IRD, APHM, MEPHI, IHU Méditerranée Infection, Faculté de Médecine et de Pharmacie, 19-21 boulevard Jean Moulin, 13385 Marseille CEDEX 05, France

**Keywords:** *Acinetobacter baumannii*, Carbapenemase, Co-occurrence

## Abstract

**Background:**

*Acinetobacter baumannii* is an important opportunistic pathogen that is rapidly evolving towards multidrug resistance and is responsible for life-threatening infections. Carbapenems are commonly used to treat *A. baumannii* infections but the emergence of carbapenemase encoding genes, such as *bla*_OXA-23-like_, *bla*_OXA-24-like,_ *bla*_OXA-58-like,_ and *bla*_NDM_ has been reported. Moreover, several studies have reported the co-occurrence of two distinct carbapenemases in some isolates. The aim of the present study is to demonstrate whether the phenomenon of co-occurrence of two distinct carbapenemase encoding genes in a single isolate still exists.

**Results:**

We studied six strains of *A. baumannii* including one harboring *bla*_OXA-23-like_ and *bla*_OXA-24-like_ genes and five with *bla*_OXA-23-like_ and *bla*_NDM_ genes. One colony of each strain was inoculated in sterile water and diluted ten-fold. Each dilution was cultivated on trypticase soy agar plates for 24 h at 37 °C and the isolated bacteria were analyzed. For two of the six tested strains, we identified two different populations of *A. baumannii,* each with a different carbapenemase, genes encoding aminoglycoside modifying enzymes, resistance phenotype, and clonal type. In addition, the two different populations had the same aspect on the agar plate.

**Conclusions:**

Here, we demonstrate that *A. baumannii* infections could be linked to multiple clones harboring different carbapenemase encoding genes in the same sample. In addition, we describe an easy method of verifying the presence of co-occurrence of carbapenemase in one isolate.

## Background

*Acinetobacter baumannii* is an important opportunistic Gram-negative bacteria pathogen that is rapidly evolving towards multidrug resistance. Worldwide, this bacterium is responsible for nosocomial infections and life-threatening infections [1]. The most common treatment is the use of carbapenems but extensive use of antimicrobial agents within hospitals has contributed to resistance against these antibiotics. The principal mechanism of resistance to carbapenems in *A. baumannii* is the production of OXA-type carbapenemases, such as OXA-23, OXA-24, and OXA-58 enzymes, and the new metallo-β-lactamase (MBL), New Delhi Metallo-β-lactamase 1 (NDM-1) [1].

The location of the carbapenemase genes is not fixed, since some studies have described *bla*_OXA-23,_ *bla*_OXA-24_ and *bla*_OXA-58_ as chromosomal or plasmidic [2]. In epidemiologic studies, co-occurrences of carbapenemase genes, such as *bla*_OXA-23_-*bla*_OXA-24,_ *bla*_OXA-23_-*bla*_NDM,_ and *bla*_OXA-58_-*bla*_NDM_ have been described [3–8]. In *bla*_OXA-23_ and *bla*_NDM_ co-expressing *Acinetobacter* spp. strains, *bla*_NDM_ can be acquired by plasmid [4, 9]. However, it remains unclear whether and how these co-existing carbapenemase genes are expressed and how they contribute to drug resistance. The aim of our study was to provide an alternative explanation for the coexistence of *bla*_OXA_ and *bla*_NDM_ genes in *A. baumannii.* For this purpose, we describe an easy method of determining whether the co-occurrence of carbapenemase genes in such bacteria was “real” or whether it is due to the existence of different bacterial clones harboring different genes in the same sample.

## Results

Ten isolated colonies from the original diluted strain were analyzed and re-identified as *A. baumannii* using MALDI-TOF MS. Original strains 519, 598, 624, and 679 and all colonies tested were positive for both *bla*_NDM_ and *bla*_OXA-23_ genes before and after the limit dilution (Table 1). We therefore considered them as “true” strains with the co-occurrence of different carbapenemase genes. Original strain 924 was positive for both *bla*_NDM_ and *bla*_OXA-23_ genes. All 10 colonies obtained after the limit dilution were tested using real time PCR: six were positive only for the *bla*_NDM_ gene while four were positive only for the *bla*_OXA-23_ gene. The original strain belonged to the sequence type ST2 as were the clones carrying the *bla*_OXA-23_ gene, whereas the clones harboring the *bla*_NDM_ gene belonged to ST25 (Table 1).

**Table 1:** Analysis of the original *A. baumannii* strains and their clones

Strain	Date of isolation (month/ year)	Sample/Ward	Carbapenemase genes	Aminoglycoside genes	ST	CTX	CAZ	TIM	TPZ	ATM	IPM	CN	TOB	AK	CIP	SXT	DO	CT
						(μg/mL)												
519 (*n* = 10)	03/2013	NA/ Intensive Care Unit	*bla*_OXA-23_- *bla*_NDM-1_	*/*	25	R (> 32)	R (> 256)	R (> 256)	R (> 256)	R (32)	R (> 32)	R (> 256)	R (> 256)	R (32)	R (> 32)	R (> 32)	R (32)	S (0.5)
598 (*n* = 10)	03/2013	NA/ Burns	*bla*_OXA-23_- *bla*_NDM-1_	*/*	25	R (> 32)	R (> 256)	R (> 256)	R (> 256)	R (32)	R (> 32)	R (> 256)	R (> 256)	R (32)	R (> 32)	R (> 32)	R (32)	S (0.5)
624 (*n* = 10)	03/2013	NA/ Intensive Care Unit	*bla*_OXA-23_- *bla*_NDM-1_	*/*	25	R (> 32)	R (> 256)	R (> 256)	R (> 256)	R (32)	R (> 32)	R (> 256)	R (> 256)	R (32)	R (> 32)	R (> 32)	R (32)	S (1)
679 (*n* = 10)	03/2013	NA/ Pediatric	*bla*_OXA-23_- *bla*_NDM-1_	*/*	25	R (> 32)	R (> 256)	R (> 256)	R (> 256)	R (32)	R (> 32)	R (> 256)	R (> 256)	R (32)	R (> 32)	R (> 32)	R (32)	S (0.5)
924 (*n* = 10)	05/2011	Pus/ Pus unit	*bla*_OXA-23_- *bla*_NDM-1_	*aph(3′)-VI*	2	R (> 32)	R (> 256)	R (> 256)	R (> 256)	R (128)	R (> 32)	R (> 256)	R (> 256)	R (> 256)	R (> 32)	R (> 32)	R (> 256)	S (1)
924-A (*n* = 4)			*bla*_OXA-23_	*aph(3′)-VI*	2	R (> 32)	R (> 256)	R (> 256)	R (> 256)	R (16)	R (> 32)	S (0.093)	S (2)	R (> 256)	R (> 32)	R (> 32)	R (> 256)	S (0.5)
924-B (*n* = 6)			*bla*_NDM-1_	*/*	25	R (> 32)	R (> 256)	R (> 256)	R (> 256)	R (128)	R (> 32)	R (> 256)	R (> 256)	R (16)	R (> 32)	R (> 32)	R (24)	S (1)
AH35 (*n* = 10)	05/2011	Urine/ Intensive Care Unit	*bla*_OXA-23_- *bla*_OXA-24_	*aph(3′)-VI*, *aac(3)-Ia, ant(2″)-I*	2	R (> 32)	R (> 256)	R (> 256)	R (> 256)	R (> 256)	R (> 32)	R (8)	R (> 256)	R (24)	R (> 32)	R (> 32)	R (> 256)	S (0.5)
AH35-A (*n* = 6)			*bla*_OXA-23_	*aph(3′)-VI*, *ant(2″)-I*	2	R (> 32)	R (> 256)	R (> 256)	R (> 256)	R (> 256)	R (> 32)	S (0.064)	S (1)	R (16)	R (> 32)	R (> 32)	R (> 256)	S (0.5)
AH35-B (*n* = 4)			*bla*_OXA-24_	*aac(3)-Ia*	2	R (> 32)	R (> 256)	R (> 256)	R (> 256)	R (128)	R (> 32)	R (8)	R (> 256)	R (24)	R (> 32)	R (> 32)	R (> 256)	S (0.5)

*CTX* cefotaxime, *CAZ* ceftazidime, *TIM* ticarcillin + clavulanic acid, *TPZ* piperacillin + tazobactam, *ATM* aztreonam, *IPM* imipenem,

*CN* gentamycin, *TOB* tobramycin, *AK* amikacin, *CIP* ciprofloxacin, *SXT* trimethoprim-sulfamethoxazole, *DO* doxycycline, *CT* colistin, *NA*, not available, *R* resistant, *S* sensitive, *ST* sequence type

**Fig. 1:**
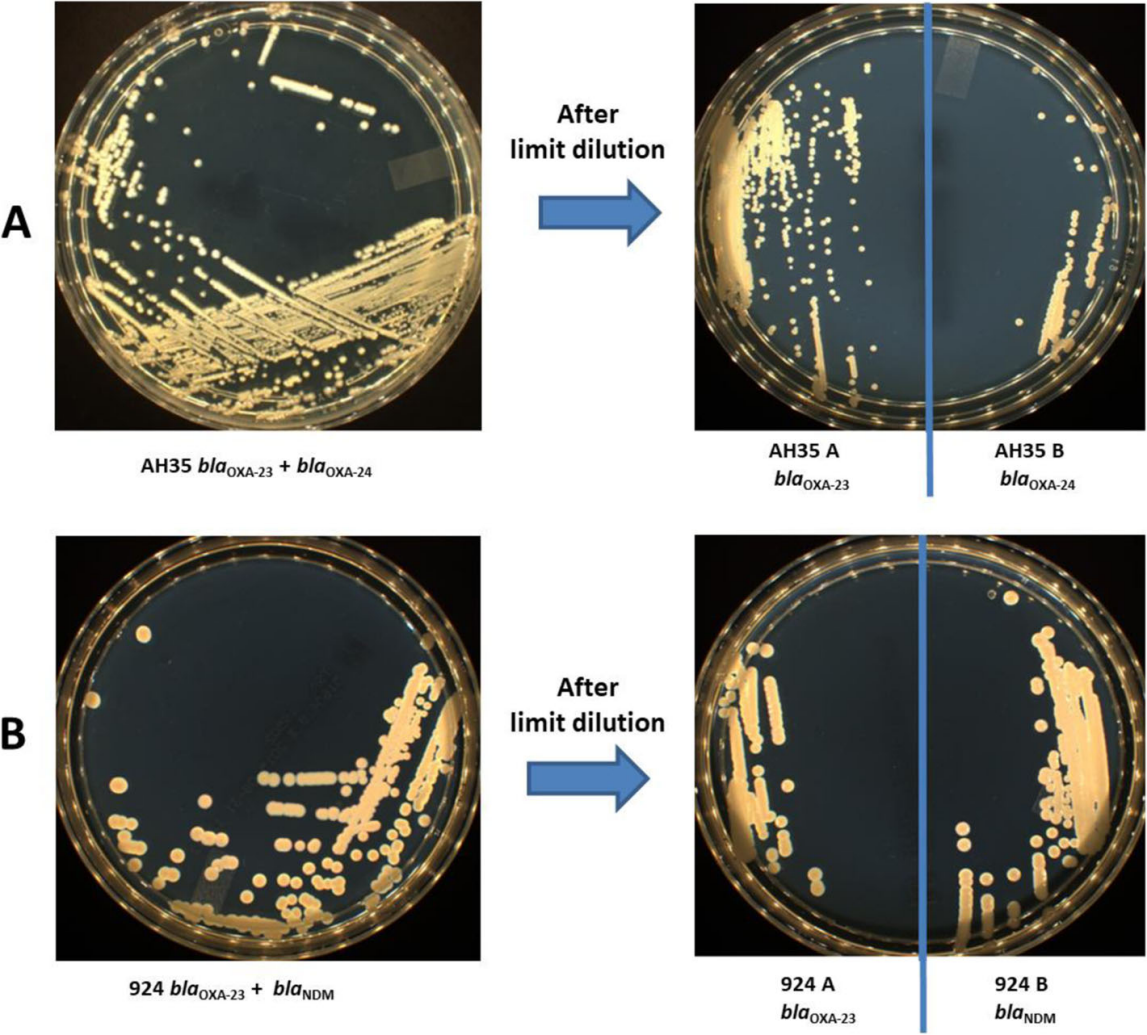
**a** Isolation of original strain AH35 *bla*_OXA-23_ and *bla*_OXA-24_ on TSA medium and isolation of clones. AH35A; *bla*_OXA-23_ on the left_,_ AH35B; *bla*_OXA-24_ on the right. **b** Isolation of original strain 924 *bla*_NDM_ and *bla*_OXA-23_ on TSA medium and isolation of clones 924A; *bla*_OXA-23_ on the left_,_ 924B and *bla*_NDM_ on the right

Original strain AH35 was positive for both the *bla*_OXA-23_ and *bla*_OXA-24_ genes_._ Of the 10 colonies which were obtained after the limit dilution, six were positive only for the *bla*_OXA-23_ gene while four were positive only for the *bla*_OXA-24_ gene_._ All strains (the original strain and the clones obtained after dilution) belonged to ST2 (Table 1). For the original strain AH35, resistance to aminoglycosides was due to the production of aminoglycoside-modifying enzymes (AMEs) *aph(3′)-VI*, *ant(2″)-I* and *aac(3)-Ia*. The clone (AH35-A) which was susceptible to gentamycin and tobramycin harbored the *bla*_OXA-23,_ *aph(3′)-VI* and *ant(2″)-I* genes whereas the clone (AH35-B) which was resistant to these antibiotics carried the *bla*_OXA-24_ and *aac(3)-Ia* genes. The original strain 924 was positive for the presence of the *aph(3′)-VI* gene. The clone (924-A) which was resistant to amikacin harbored the *bla*_OXA-23_ and *aph(3′)-VI* genes; while no AME genes were found in the clone (924-B) carrying the *bla*_NDM_ gene and resistant to amikacin with relatively low MIC. Original strains AH35 and 924 were chimeras of their two clones. The results of the antibiotic susceptibility testing (AST) of the obtained clones after the limit dilution revealed two different resistance phenotypes according to the aminoglycosides presented in the different clones (Table 1). After sub-culturing the two original strains (924 and AH35) in the same conditions and the same TSA medium (Fig. 1), we observed no phenotypic differences in terms of size, form or color.

## Discussion

Since *A. baumannii* is able to remodel its genome, antibiotic use and the host environment might impose selective forces that drive its rapid adaptive evolution. Microscale genome modification has been revealed through the analysis of single nucleotide polymorphisms (SNPs) between *A. baumannii* strains isolated from the same patient. This modification can lead the emergence of resistance and to different sequence typing by modifying a single allele [10]. However, this explanation cannot be applied to our case, because ST2 and ST25 have only two common alleles. Mutations in five alleles would be a substantial evolution. Moreover, this cannot be an evolution of a clone because the presence of different carbapenemase-encoding genes was also observed.

Another phenomenon, referred to as small colony variants (SCVs), mostly described in *Staphylococcus aureus,* was also identified from one sample with different susceptibility to antibiotics from the parent strain [11]. SCVs can cause latent and recurrent infections and have been observed in many genera of bacteria, including Gram-negative bacteria such as *Pseudomonas aeruginosa* [12]. In our case, the populations are morphologically identical and do not have the aspect of small colony variants.

The presence of different populations of a same species in the same sample had been already described in *S. aureus* [13]. Similarly to our study, different clones with different resistance phenotypes, such as methicillin-susceptible and methicillin-resistant *S. aureus* strains could be isolated from the same sample by increasing the number of colonies tested for each sample [14].

*Bla*_OXA-23_, *bla*_OXA-24_ and *bla*_NDM_ can be located either on the chromosome or plasmid. For this reason, we tend to believe that co-occurrence with other resistance genes is due to the presence of different genes in the same strain. However, another explanation could be the presence of a polyclonal population of *A. baumannii* from the same sample. These populations are morphologically undetectable with the naked eye. Although the number of strains studied was limited, our work represents the proof of concept that co-occurrence of carbapenemases in *A. baumannii* could be due to multiple clones.

## Conclusion

In this work, we demonstrated that from one sample and from an original “chimera” strain with two carbapenemase genes which were highly resistant to antibiotics, we were able to isolate two less resistant strains with only one gene encoding for carbapenems resistance. The presence of different clonal types and different genes encoding aminoglycoside modifying enzymes in each clone is also evidence of polyclonal coexistence in a single sample.

Another option, in similar case to this current study, is the coexistence in a single sample of different clones harboring one carbapenemase encoding gene as well as different resistance genes. In addition, this study has strong implications for clinical practice. The use of only one colony from one sample to conduct AST may omit another population which may be more resistant.

## Methods

Six *A. baumannii* strains (924, 519, 598, 624, and 679) isolated from two Algerian hospitals were studied; five *A. baumannii* co-expressed *bla*_OXA-23_ and *bla*_NDM_ genes recovered from a hospital in Algiers, and one *A. baumannii* (AH35) co-expressed *bla*_OXA-23_ and *bla*_NDM_ gene isolated from a urine sample in an intensive care unit in a hospital in Setif (Table 1). These clinical isolates were taken for the purpose of this study. Identification was confirmed using matrix-assisted laser desorption and ionization time-of-flight mass spectrometry (MALDI-TOF) (Microflex, Brüker Daltonics, Bremen, Germany) [15].

**Fig. 2:**
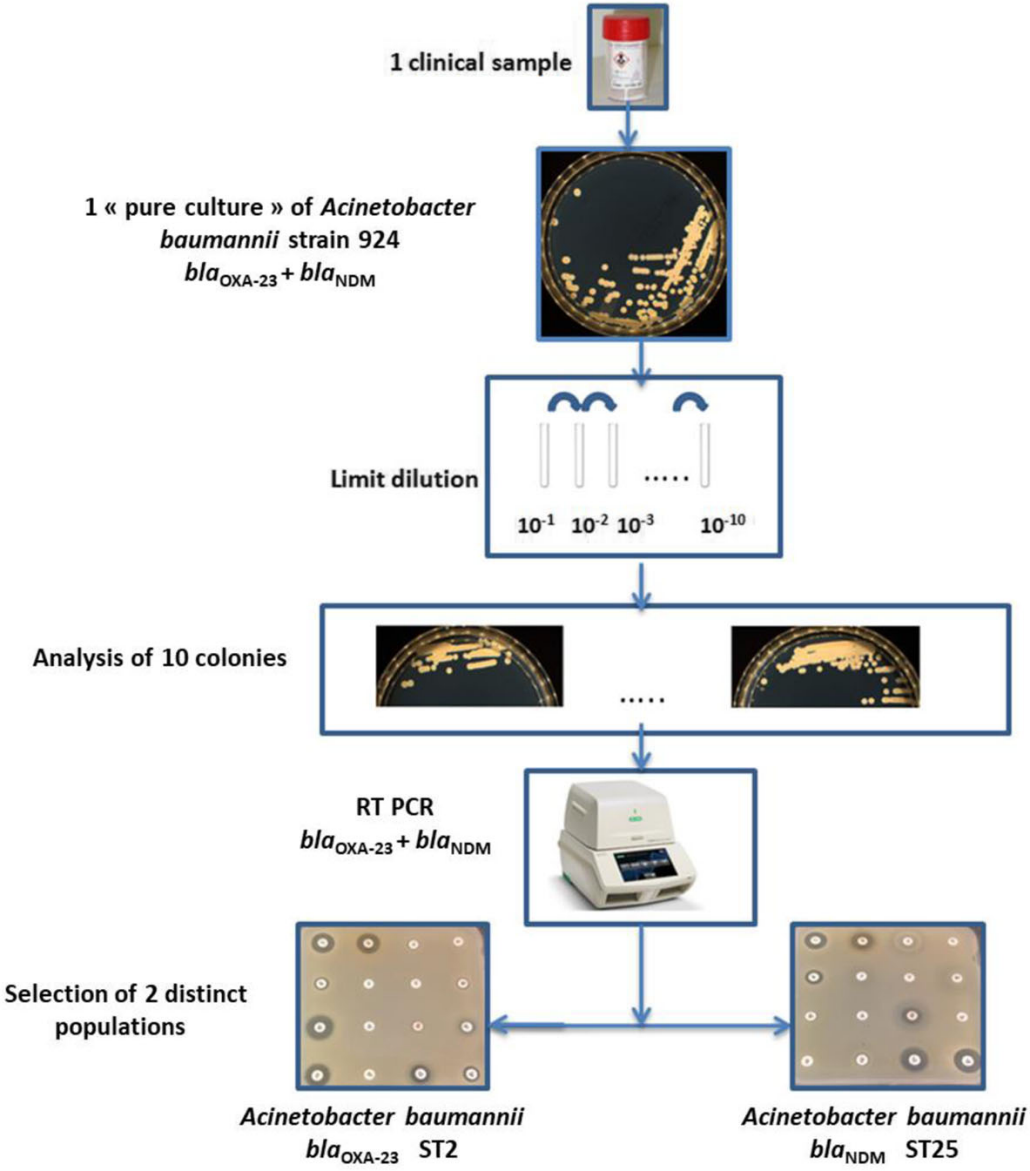
Example of the study of the strain 924

For each strain, one colony was inoculated in sterile water and diluted ten-fold to isolate a single clone. Each dilution was cultivated on Trypticase Soy Agar (TSA) plates for 24 h at 37 °C and isolated bacterial were analyzed. Antibiotic susceptibility was determined by minimum inhibitory concentrations (MICs) using broth microdilution method (Biocentric) for colistin and using the E-test method (bioMérieux) for others antibiotics tested in accordance with the European Committee on Antimicrobial Susceptibility Testing (EUCAST). Real time PCR was performed to verify the presence of the *bla*_OXA-23,_ *bla*_OXA-24_ and *bla*_NDM_ genes [16, 17]. Standard PCR amplification was carried out using primers for amplification of the *aph(3′)-VI*, *ant(2″)-I*, *aac(3)-Ia*, *aac(6′)-Ib* and *armA* genes [18]. Clonal types of the isolates were determined using multilocus sequence typing (MLST) in line with the Pasteur schemes (https://pubmlst.org/abaumannii/). Each stage of the materials and methods section is summarized in Fig. 2.

## Abbreviations

AME: Aminoglycoside-modifying enzyme
AST: Antibiotic susceptibility testing
EUCAST: European Committee on Antimicrobial Susceptibility Testing
MALDI-TOF: Matrix-assisted laser desorption and ionization time-of-flight mass spectrometry
MBL: Metallo-β-lactamase
MIC: Minimum inhibitory concentration
MLST: Multilocus sequence typing
SCV: Small colony variants
SNP: Single nucleotide polymorphism
TSA: Trypticase soy agar
